# Electronic identification keys for species with cryptic morphological characters: a feasibility study using some *Thesium* species

**DOI:** 10.3897/phytokeys.172.53484

**Published:** 2021-02-16

**Authors:** Natasha Lombard, Margaretha Marianne le Roux, Ben-Erik van Wyk

**Affiliations:** 1 Biosystematics Research and Biodiversity Collections Division, South African National Biodiversity Institute, Private Bag X101, Pretoria, 0001, South Africa University of Johannesburg Johannesburg South Africa; 2 Department of Botany and Plant Biotechnology, University of Johannesburg, PO Box 524, Auckland Park, 2006, South Africa South African National Biodiversity Institute Pretoria South Africa

**Keywords:** Best practice, interactive key, key construction, photographic key, Santalaceae, South African plants, taxonomic impediment, Xper^3^

## Abstract

The popularity of electronic identification keys for species identification has increased with the rapid technological advancements of the 21^st^ century. Although electronic identification keys have several advantages over conventional textual identification keys and work well for charismatic species with large and clear morphological characters, they appear to be less feasible and less effective for species with cryptic morphology (i.e. small, obscure, variable characters and/or complicated structures associated with terminology that is difficult to interpret). This is largely due to the difficulty in presenting and illustrating cryptic morphological characters unambiguously. When taking into account that enigmatic species with cryptic morphology are often taxonomically problematic and therefore likely exacerbate the taxonomic impediment, it is clear that species groups with cryptic morphology (and all the disciplines dependent on their correct identification) could greatly benefit from a user-friendly identification tool, which clearly illustrates cryptic characters. To this end, the aim of this study was to investigate and develop best practices for the unambiguous presentation of cryptic morphological characters using a pilot interactive photographic identification key for the taxonomically difficult plant genus *Thesium* (Santalaceae), as well as to determine its feasibility. The project consisted of three stages: (1) software platform selection, (2) key construction and (3) key evaluation. The proposed identification key was produced with Xper^3^ software and can be accessed at http://www.xper3.fr/xper3GeneratedFiles/publish/identification/1330098581747548637/mkey.html. Methodologies relating to amongst others, character selection and delineation, visual and textual descriptions, key construction, character coding and key evaluation are discussed in detail. Seventeen best practices identified during this study are subsequently suggested for future electronic key compilation of species with cryptic morphology. This study indicates that electronic identification keys can be feasible and effective aids for the identification of species with cryptic morphological characters when the suggested best practices are followed.

## Introduction

Species identification underpins the majority of biological sciences (Stevenson et al. 2003; Farr 2006; Farnsworth et al. 2013). Attributing a name to a specimen is central to, amongst others, the classification of groups of organisms, ecology, species and habitat conservation, ecological restoration and the management of biological collections (Lawrence and Hawthorne 2006; De Carvalho et al. 2007; Joly et al. 2014; Joly et al. 2019). Traditionally, textual dichotomous keys have been the main tools used for species identification (Walter and Winterton 2007; Nimis et al. 2012; Seo and Oh 2017). More recently, rapid technological advancements of the 21^st^ century have resulted in the production of a wide array of electronic identification guides (Stevenson et al. 2003; Farnsworth et al. 2013) that range from simple textual electronic dichotomous keys (e.g., Leistner 2000; Beuk 2019) to interactive mobile identification applications (apps) with access to large multimedia databases (e.g., De Vaugelas et al. 2011; Merlin Bird ID App, https://merlin.allaboutbirds.org/) and automatic visual recognition apps (e.g., Zhao et al. 2015; PlantSnap, https://www.plantsnap.com/). Electronic identification keys have become commonplace (e.g., Kirchoff et al. 2011; Nimis et al. 2012; Seo and Oh 2017; Jouveau et al. 2018; Bodin et al. 2019; Reeb and Gradstein 2020), are relatively easy to produce and are aimed at enhancing the accessibility and usability of identification keys, as well as the efficiency and accuracy of identifications (Drinkwater 2009; Kirchoff et al. 2011).

Although electronic identification keys have several advantages over conventional identification keys [as detailed in Farr (2006) and Dallwitz et al. (2000)], the few studies comparing the performance of these different identification keys have showed mixed results. Stagg et al. (2015) showed that the accuracy and speed of woodland moss identification was higher using a traditional dichotomous key than an electronic key, while Seo and Oh (2017) found that orchid species were more accurately identified by senior college students when using an electronic identification key than when using a textual dichotomous key or a guide book based on flower colour. Stagg and Donkin (2017) showed that identifications of United Kingdom (UK) wild flowers were significantly more accurate using an electronic app than a guide book, but that the identification accuracy of UK winter trees was significantly lower when using an electronic app than when using guide books. Stagg and Donkin (2017) posited two reasons for their contrasting results. First, the number of tree species was less than the number of wild flowers so that browsing for tree species in the printed guides was more time efficient than browsing for wild flowers of which there are many species (Drinkwater 2009; Stagg and Donkin 2017). Second, winter tree character states were perceived as subjective, ambiguous and overall cryptic compared to wild flower character states which were clear and concise (Stagg and Donkin 2017). These comparative studies indicate that while electronic identification keys such as interactive photographic keys are effective when identifying charismatic species with large and clear morphological characters, they are often ineffective when identifying enigmatic species with cryptic morphological characters. Here cryptic characters (not to be confused with cryptic species) refer to any morphological character which might cause uncertainty or confusion during the identification process due to one or a combination of the following: (1) very small size [e.g., characteristics of leaf margins and venation in mosses (Stagg et al. 2015); minute characters of armoured scale insects (Schneider et al. 2019)], (2) obscure nature [e.g., subtle differences in bud colour of winter trees (Stagg and Donkin 2017); metasternum related characters in some parasitoid wasps (Klimmek and Baur 2018)], (3) intra-specific variation [e.g., flower colour variation in the carnivorous plant genus *Drosera* L. (Drinkwater 2009); pronotum colour variation in ladybirds (Jouveau et al. 2018)], and (4) complicated structures associated with terminology that is difficult to interpret [e.g., inflorescences of grasses (Fish et al. 2015); thorax morphologies of Brazilian sand flies (Rocha et al. 2019)]. The challenge remains to determine which aspects are critical to produce electronic identification keys that can successfully identify species with cryptic morphological characters.

Enigmatic species with cryptic characters such as many plants, insects, bryophytes and microorganisms are common and are often surrounded by much taxonomic uncertainty (Convention on Biological Diversity 2007). This is partly due to a research bias towards charismatic species and partly due to the difficulty in finding and describing characters with which to delimit and identify enigmatic species. Often only one or a few specialist taxonomists can accurately identify them. All of these aspects add to the taxonomic impediment (Convention on Biological Diversity 2007; Walter and Winterton 2007; Dar et al. 2012) and it is clear that species groups with cryptic characters (and all the disciplines dependent on their correct identification) could greatly benefit from a user-friendly identification tool that clearly illustrates cryptic characters. To address this need we investigated the use of a multi-access interactive photographic identification key as an identification aid for selected species of the morphologically difficult and near cosmopolitan genus *Thesium* L. [Santalaceae (The Angiosperm Phylogeny Group 2016)].

*Thesium* is a hemi-parasitic plant genus of ± 350 species that has its centre of diversity in southern Africa, with ± 175 species (Lombard et al. 2020). Some *Thesium* species are of economic importance (Lombard et al. 2020). For instance, *T. humile* Vahl has caused substantial losses to cereal crops in the Mediterranean region (Belakhdar et al. 2014), while *T. chinense* Turcz. is sold commercially in Asia as an herbal medicine to treat a wide array of ailments (Lombard et al. 2020). Species of this genus are notoriously difficult to identify due to, amongst others, the extreme intra-specific variation observed in vegetative morphology, as well as their diminutive flowers (< 10 mm) which contain several important diagnostic characters (Hill 1915). Identifications are further complicated by the large number of species in the genus and the superficial similarities among species (Hill 1915). Current identification keys for South African *Thesium* species are textual keys (e.g., Hill 1925) that are often very difficult to use due to the overlap of character states between couplets (to account for intra-specific variability), as well as the difficulty in describing subtle differences in the general impression, size and shape (GISS) of species (García et al. 2018). *Thesium* is therefore an ideal group in which to study cryptic characters and their representation in an electronic identification key.

The aim of this study was to investigate and develop best practices for the unambiguous presentation of cryptic morphological characters using a pilot interactive photographic identification key. The project was developed by (1) identifying practical, easy-to-use software with which to construct a photographic identification key, (2) producing a pilot identification key for 25 *Thesium* species found in the eastern part of South Africa and (3) evaluating the effectiveness of the identification key with a target group of users from different backgrounds. We subsequently propose a multi-access interactive photographic identification key produced with Xper^3^ software.

## Materials and methods

### Taxa

As the intent of this study was to investigate and demonstrate principles behind the unambiguous presentation of cryptic characters and not to produce a comprehensive field-ready identification key, a subset of 25 species (Table 1) from the morphologically difficult genus *Thesium* were selected as a case study. These species are among ± 60 *Thesium* species that occur in the eastern part (summer rainfall area) of South Africa and were chosen, firstly because they have been observed, collected and photographed by the authors in their living state and natural habitat. Information and media collected in the field is advantageous when constructing photographic identification keys and circumvents several problems associated with electronic key construction from literature and preserved collections (see Morse et al. 1996; Drinkwater 2009). Second, the majority of the 25 species are notoriously difficult to identify as is evidenced by the numerous identification queries the authors received, as well as by the mixed specimen collections encountered in several South African herbaria. This indicates that even trained taxonomists responsible for curating these collections had considerable difficulty in identifying the species in question. Third, recent (and ongoing) taxonomic studies of 12 of the 25 species (Mashego and le Roux 2018; Visser et al. 2018; Lombard et al. 2019, Lombard et al. in prep.) prompted the compilation of user-friendly identification keys and a platform for information dissemination to non-taxonomist users. Fourth, the identification key contributes to research on *Thesium* that is considered a high priority for taxonomic research in South Africa (Victor et al. 2015; Victor 2020).

**Table 1.** The 25 *Thesium* species included in the pilot interactive photographic identification key, as well as the most recent taxonomic treatment for each species.

**Species**	**Taxonomic treatment used**
*Thesium angulosum* A.DC.	Hill 1925
*Thesium asterias* A.W.Hill	Hilliard 2006
*Thesium confine* Sond.	Mashego and le Roux 2018
*Thesium costatum* A.W.Hill	Hill 1925
*Thesium cupressoides* A.W.Hill	Hill 1925
*Thesium davidsoniae* Brenan	Brenan 1985
*Thesium durum* Hillard & B.L.Burtt	Mashego and le Roux 2018
*Thesium goetzeanum* Engl.	Visser et al. 2018
*Thesium gracilarioides* A.W.Hill	Visser et al. 2018
*Thesium gracile* A.W.Hill	Visser et al. 2018
*Thesium gypsophiloide*s A.W.Hill	Visser et al. 2018
*Thesium impeditum* A.W.Hill	Hill 1925
*Thesium magalismontanum* Sond.	Hill 1925
*Thesium multiramulosum* Pilg.	Hilliard 2006
*Thesium natalense* Sond.	Lombard et al. in prep.
*Thesium ovatifolium* N.Lombard & M.M.le Roux	Lombard et al. 2019
*Thesium pallidum* A.DC.	Hill 1925
*Thesium procerum* N.E.Br.	Visser et al. 2018
*Thesium racemosum* Bernh.	Hill 1925
*Thesium resedoides* A.W.Hill	Visser et al. 2018
*Thesium scirpioides* A.W.Hill	Lombard et al. in prep.
*Thesium transvaalense* Schltr.	Hill 1925
*Thesium utile* A.W.Hill	Hill 1925
*Thesium vahrmeijeri* Brenan	Visser et al. 2018
*Thesium zeyheri* A.DC.	Hill 1925

### Software platform: Xper^3^

Xper^3^ was chosen as the platform for the present study as it is a free access self-controlled programme where no external data storage or servers are needed, and which includes all of the functionalities required by the authors (e.g., multi-access keys, visual and text descriptors and species profiles) (Vignes-Lebbe et al. 2016; Vignes-Lebbe et al. 2017; Pinel et al. 2017). The platform allows for remote access, is intuitive and user friendly for both authors and users, allows for multiple contributors (including concurrent data editing), has the option for both web-based and mobile interfaces and can function as a taxonomic data management programme. The Xper^3^ home page can be accessed at http://www.xper3.fr/ and detailed user documentation at http://wiki.xper3.fr/lib/exe/fetch.php?media=wiki:xper3documentation.pdf.

### Key construction

Construction of the identification key was completed in four steps: 1) data collection, 2) taxonomic and character backbone construction, 3) character coding and 4) species profile compilation.

### Data collection

**Characters and character states used**

A total of 26 characters were used in the key (Table 2), including 24 discrete characters and two range characters. In the absence of a published or widely accepted list of morphological characters for the genus, morphological characters and character states were adapted from an unpublished character list by, and initial discussion with, Daniel Nickrent (pers. comm.). All informative vegetative and reproductive characters that could clearly be shown with photographs were included. The maximization of the number of morphological characters available to choose from facilitates use by both non-specialist and specialist users. The majority of morphological characters included can be observed with the naked eye, but a 10x hand-lens or light microscope is needed for some of the diminutive floral characters such as style length, stigma position and placental column shape. Character states were delineated and presented in such a way as to facilitate unambiguous interpretation by users and following the guidelines provided in Walter and Winterton (2007); also see Results and Discussion. Each of the provinces of South Africa were also included as characters in the key (see Table 2), as this proved to be the most efficient and user-friendly way to account for the geographical distribution of each species.

**Images**

Live material was photographed in the field during the flowering seasons (September to February) of 2016, 2017 and 2018 using a Canon EOS 400D camera and Canon EF 100 mm/2.8 USM macro lens. Where live material could not be accessed for certain characters or species, photographs of herbarium material were used. One of the advantages of electronic identification keys is that they can continuously be updated and current images can be replaced with superior images as they become available. Flowers from herbarium material were rehydrated by placing them in Windowlene (cleaning agent) for 15 min before being photographed. Herbarium material were photographed with standard smartphone cameras (Huawei P9 lite, Samsung S7) by aiming the smartphone camera lens at the eyepiece of a light microscope (Nikon SMZ 745 T stereo microscope, Nikon Corporation) so that the enlarged image becomes visible through the eyepiece and then taking the photo. Photographs were later edited where necessary to enhance characters using Microsoft PowerPoint software v. 14.0.7229.5000 (Microsoft Corporation). All photographs included here and in the key were taken by the authors unless stated otherwise. Bract shape and placental column shape photographs were supplemented with illustrations to ensure unambiguity (Leggett and Kirchoff 2011).

**Table 2.** The 26 characters and their respective character states used to distinguish between selected *Thesium* species in a pilot interactive photographic identification key. Definitions of characters and character states are given in the identification key (http://www.xper3.fr/xper3GeneratedFiles/publish/identification/1330098581747548637/mkey.html).

Character	Character state							
	1	2	3	4	5	6	7	8
Distribution in South Africa (Province)	Eastern Cape	Free State	Gauteng	KwaZulu-Natal	Limpopo	Mpumalanga	Northwest	Northern Cape
Habit 1 (shape)	Erect	Virgate	Decumbent or procumbent					
Habit 2 (woodiness)	Woody	Herbaceous						
Habit 3 (branching position)	Unbranched	From the lower third	From the middle third	From the upper third				
Root system	Branched	Underground stem						
Vegetative scales	Present	Absent						
Plant height	(Actual measurement in m)							
Stem cross-section	Smooth	Ribbed (sulcate)	Winged (alate)					
Plant hairiness (indumentum)	Hairs absent (glabrous)	Hairs present (pubescent)						
Foliage type	Leaves	Scales						
Leaf orientation	Appressed	Spreading	Not applicable					
Leaf attachment	Fused to stem (decurrent)	Not fused to stem (not decurrent)						
Inflorescence 1 - apex	Indeterminate	Determinate						
Inflorescence 2 - structure	Raceme-like	Cymes	Spike-like	Solitary				
Inflorescence 3 - synflorescence flower arrangement combinations	Monochasium	Dichasium	Not applicable					
Flower shape	Cup-shaped (stellate/ patelliform)	Bell-shaped (campanulate)	Tubular					
Involucral bracts	Absent	Present						
Bract fusion to flower stalk (bract recaulescence)	Not fused	Partially fused	Fully fused	Not applicable				
Bract shape	Lanceolate	Linear	Ovate	Deltoid				
Corolla lobe shape	Triangular	Linear						
Flower disc	Present	Absent						
Corolla lobe margin hairiness (indumentum)	Dense hairs	Sparse hairs	Lacinulate	Papillose (ciliate or erose)	Smooth (glabrous)			
Style length	Sessile	Short	Long					
Stigma position	Below the anthers	In line with the anthers	Above the anthers					
Placental column shape	Straight	Twisted						
Fruit length	(Actual measurement in mm)							

### Taxonomic and character backbone construction

**Taxonomic backbone**

**Figure 1.**
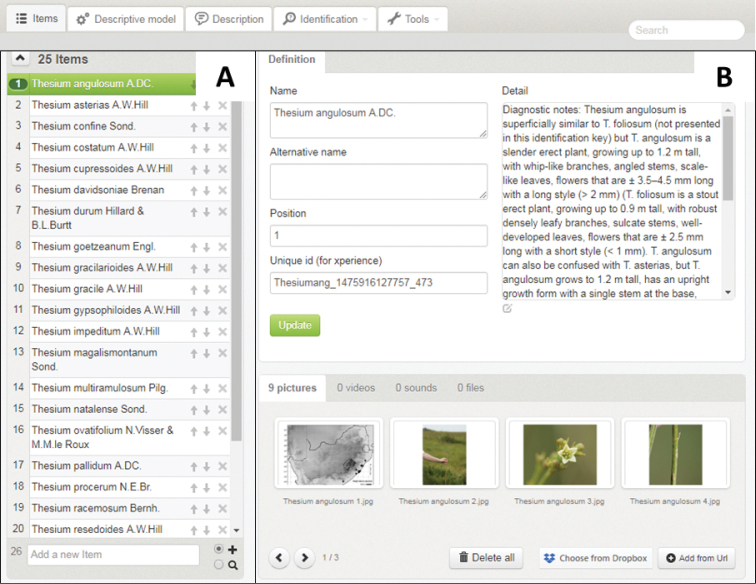
Xper^3^ author interface showing **A** the list of 25 *Thesium* species (items) which forms the taxonomic backbone of the interactive photographic identification key, as well as **B** an example of the supplementary information provided for *T. angulosum*.

The first data to be uploaded into Xper^3^ were the scientific names of the 25 *Thesium* species included in this study (Fig. 1; Table 1). Sound taxonomy is a crucial pre-requisite for identification key construction. Therefore, species concepts and names were taken from the most recent taxonomic treatments of each species, which are provided in Table 1.

**Character backbone**

**Figure 2.**
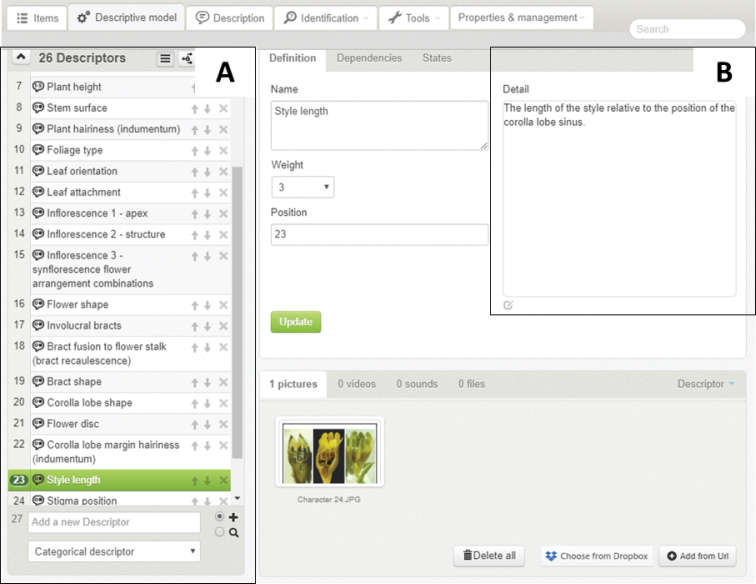
The Xper^3^ author interface showing **A** the list of 26 characters (descriptors) which forms the character backbone of the interactive photographic identification key, as well as **B** an example of the supplementary information provided for the character, style length.

After the taxonomic backbone was completed the character backbone was compiled. This was done by adding each character and its corresponding character states into Xper^3^. Each character and character state was listed in the key using descriptive terms such as flower shape, style length etc. (e.g., Fig. 2). Generalist terminology was sometimes used and specialist terminology added in brackets where applicable to cater for specialist users. Where needed, terminology was supplemented with textual descriptions further explaining what was being shown (e.g., Fig. 2).

**Figure 3.**
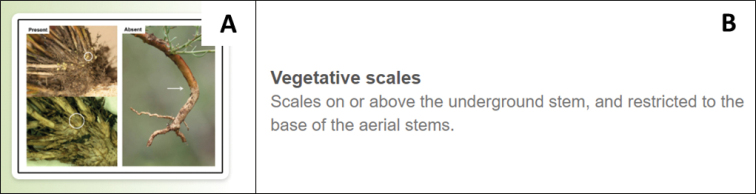
An example of the visual and textual presentation of a character, vegetative scales, in the user interface of the interactive photographic identification key. **A** representative images of each character state (present and absent) of the character, with the relevant structures further emphasized using circles and arrows **B** a textual description of the character.

**Figure 4.**
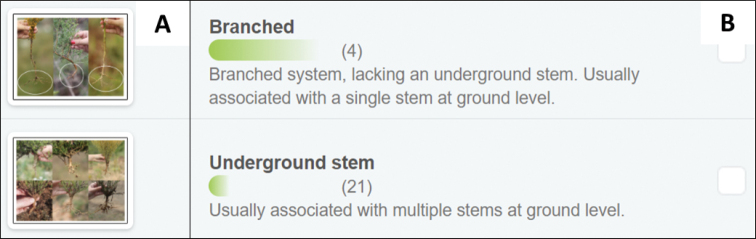
An example of the visual and textual presentation of character states in the user interface of the interactive photographic identification key. For the character, root system, each character state (branched and underground stem) is **A** illustrated with multiple photographs to show variation, as well as **B** a textual description.

In addition to terminology and textual descriptions, each character and character state was also visually represented with a figure plate containing representative photographs. For example, the character vegetative scales, was illustrated with three photographs; two plants with vegetative scales and one without vegetative scales (Fig. 3). Where possible, each character state was illustrated with multiple photographs to enhance clarity. For example, the branched character state of the root system character was illustrated with three photographs and the underground stem character state with six photographs (Fig. 4). Due to the small and cryptic nature of many morphological structures, relevant characters and/or character states were highlighted in certain images, either by a circle or an arrow. Individual images were labelled where needed for clarity. All figure plates were compiled in Microsoft PowerPoint software v. 14.0.7229.5000 (Microsoft Corporation), exported as JPEG files, resized to a standard height of 1000 pixels using FastStone Photo Resizer 3.8 software (FastStone Soft), saved to Dropbox and uploaded into Xper^3^ by copying the Dropbox link for each figure plate to the “Add from Url” feature in Xper^3^. All photographs and figures were compiled based on the best practices provided by Leggett and Kirchoff (2011).

### Character coding

**Figure 5.**
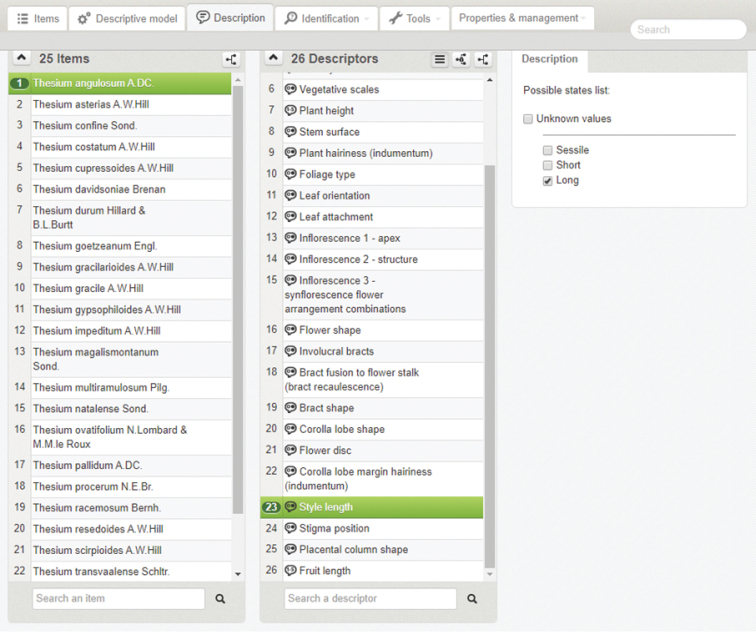
The Xper^3^ author interface showing an example of character state coding in the interactive photographic identification key, where the style length of *Thesium angulosum* is coded as long.

After constructing the taxonomic- and character backbones of the key, character states were manually coded for each species in Xper^3^, for example, the style length of *T. angulosum* is long (Fig. 5). The appropriate character states for each species were determined by the authors through examining species in the field, as well as studying herbarium material at the National Herbarium in Pretoria (PRE), South Africa. Subsequent knowledge gaps were filled using the most recent taxonomic description available for each species (Table 1). It was occasionally necessary to select more than one character state for a species to account for the intra-specific variation observed in *Thesium* species, as well as differences in user interpretation (see Results and Discussion). Character weighting was not utilized in this study.

### Species profiles

**Figure 6.**
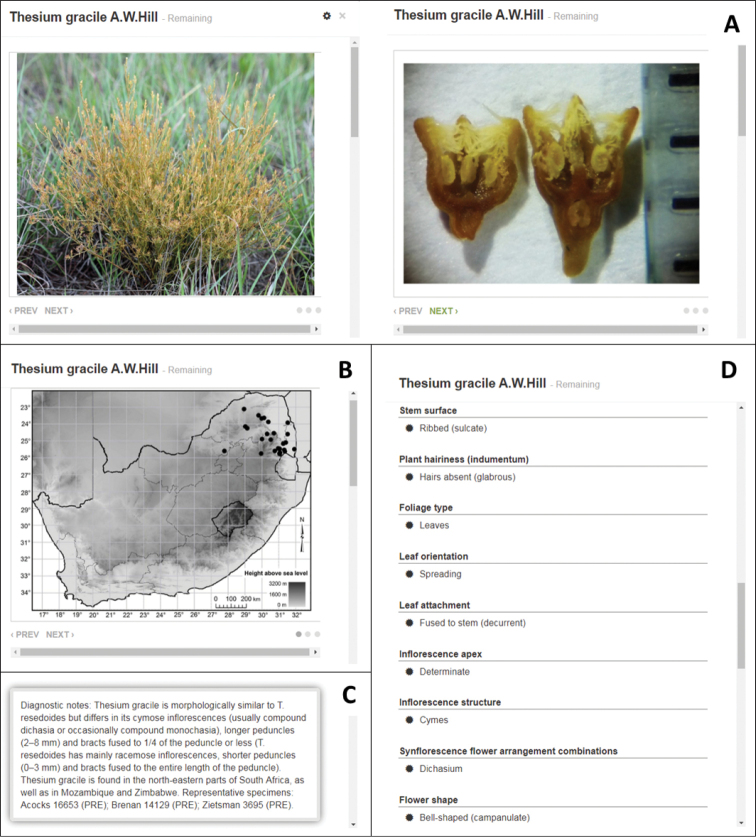
An example of a species profile with supplementary information in the user interface of the interactive photographic identification key, showing **A** representative photographs, **B** a distribution map, **C** diagnostic notes and **D** a list of character states of *Thesium gracile*.

The final step in key construction was to create a profile for each species (Fig. 6) that includes contextual photographs, a detailed distribution map, a short diagnosis and a list of character states for that species (automatically generated by Xper^3^). Photographs included here show important diagnostic characters, as well as other general impressions of each species (e.g., the habit and flowers) to aid identification. The short diagnoses give notes on separating morphologically similar species. Where needed, comparisons with similar species not presented in the key were also included to ensure correct identifications despite the nature (pilot study) of the key.

### Key evaluation

**Target group testing**

A target group testing was done at a Plant Specialist Group meeting at Buffelskloof Nature Reserve, South Africa. The 22 participants included amateur botanists, conservationists, ecologists, environmental consultants, horticulturists and taxonomists. Participants were divided into six groups of three or four and provided with access to the Xper^3^ key on their smartphones and laptops, a printed dichotomous key (automated by the Xper^3^ platform), a microscope and a flower dissecting kit (a razor blade, tweezers and dissecting needle). Each group was given ample fresh material of three *Thesium* species (*T. confine* Sond., *T. procerum* N.E.Br. and *T. utile* A.W.Hill) and was asked to identify them accurately at their own pace. During the exercise participants provided feedback which allowed characters, character states, terminology and photographs that caused confusion and/or uncertainty to be identified. Participants also gave general feedback on the usability of the key and all of these suggestions were incorporated into an improved key (discussed below). The authors aim to continuously improve the key by trial and revision and also expand the key by systematically adding more *Thesium* species.

**Checkbase**

In addition to the target group testing, the identification key was also evaluated using Checkbase, a build-in tool provided by Xper^3^. Checkbase provides information on discrimination between (1) items (species), (2) descriptions (characters) and (3) character states, as well as (4) missing character states.

## Results and discussion

In the current age of information and digital technology more emphasis is being placed on the development of electronic resources to advance the identification of species, which is vital for all practices related to or dependent on biological studies (Walter and Winterton 2007; Kirchoff et al. 2011). One of the remaining challenges in electronic identification key development, namely the effective presentation of cryptic morphological characters to ensure successful identifications in morphologically difficult species groups, was addressed in a pilot study using a *Thesium* identification key. The identification key is accessible through the following link: http://www.xper3.fr/xper3GeneratedFiles/publish/identification/1330098581747548637/mkey.html.

### Software platform selection

While Xper^3^ software provided a pragmatic platform for key construction in this study, the general principles and best practices discussed hereafter, can be applied to any software with the relevant functionalities (e.g., DELTA, http://www.delta-intkey.com/; Lucid, http://www.lucidcentral.org/). Walter and Winterton (2007), Drinkwater (2009) and Dallwitz et al. (2000) amongst others provide summaries of the general advantages of interactive keys, which are applicable to all electronic keys but not the specific focus of this study.

### Multi-access keys

Software platforms with a multi-access approach, where a user can choose any of the available characters throughout the key, circumvent multiple problems associated with the identification of species, especially those with cryptic characters (Walter and Winterton 2007; Drinkwater 2009). Compared to single-access keys, where characters follow on each other in a predetermined order, multi-access keys allow users to select the characters that are available and that they are most confident about, thereby optimizing identification accuracy (Morse et al. 1996; Drinkwater 2009). It also decreases the chances of a user abandoning the identification process altogether due to the cryptic nature of some characters (e.g., a minute ovary that can only be accessed by dissecting a flower) or guessing character states, which might lead to misidentifications (Morse et al. 1996).

Multi-access keys furthermore cater for the unambiguous presentation of cryptic characters and character states by allowing authors to utilize numerous character divisions. For example, the inflorescence structure of *Thesium* species is an important distinguishing character, but often varies considerably and is notoriously difficult to interpret. Its incorporation into traditional textual keys (Hill 1915, 1925) has resulted in several overlapping character states between divisions, the use of vague phrases such as “more or less” and other complex terminology. When taking into account that inflorescence type is only the second division in Hill’s key, it is understandable that users have struggled to identify *Thesium* species correctly. In contrast, the electronic key proposed here clearly delineates different inflorescence types using three characters and nine character states with no overlap and also illustrates each division with both visual (including multiple photographs) and text aids.

### Updatable keys

Software allowing for updates and changes to be made to identification keys after publication is pertinent for species groups with cryptic morphology as these groups are likely to be taxonomically problematic and subject to ongoing taxonomic study. For example, subsequent to the construction of the identification key presented here, a *Thesium* species new to science was described (Lombard et al. 2019) and a taxonomic revision of two species in the key completed (Lombard et al. in prep.). Information from both these studies was easily incorporated into the database in Xper^3^. The addition of new species to electronic keys is especially important in enigmatic species groups with cryptic characters as the electronic key might be one of the only user-friendly information sources available to non-specialist users. Furthermore, minimizing the lag time between taxonomic research and its availability to the end user, for example through an identification key, might contribute somewhat to alleviating the taxonomic impediment (Walter and Winterton 2007). In addition to research, user feedback and its incorporation is central to a study like the one presented here as user experience is the ultimate measure of both the success of cryptic character presentation and species identifications, and allows for continual improvement of the identification key.

### Species profiles

Species profiles with supplementary information and media on each species form part of many software platforms and contribute considerably to accurate identifications (Kumar et al. 2012). Xper^3^ species’ profiles include, amongst others, contextual photographs, detailed distribution maps and short diagnoses, and can easily be accessed at any point in the identification process. In dealing with species with cryptic morphology, it is recommended that users follow the key until they are uncertain about all of the remaining characters. If more than one species remains, the profiles of the remaining species should be consulted for a final identification (one can flip from one profile to the next in Xper^3^) (Drinkwater 2009). Detailed distribution maps are also very useful as they are unambiguous and instantly allow a user to determine whether the species in question occurs in the applicable geographical area. Furthermore, the value of contextual photographs displaying the general impression, size and shape (GISS) of a species should not be overlooked. While two species might differ in only one or two particularly cryptic characters, they are often easily distinguishable by their GISS. “A picture is worth a thousand words” and relays information which is difficult to capture in words. For species with cryptic morphology, photographs are the crux of resolving confusion originating from traditional textual identification keys. Lastly, the short diagnosis provided for each species further streamlines the identification process by providing information on similar species and how they differ from one another (Stevenson et al. 2003). Species profiles can also be used independently of the identification key to confirm species identities or for additional information on a particular species.

### Key construction

**Character and character state delineation**

In this study, maximizing the number of valuable characters while minimizing the number of associated character states proved most pragmatic. Contrary to species groups with clearly defined morphological characters (e.g., Jouveau et al. 2018), maximizing the number character options in morphologically difficult groups provides more opportunities for users to select characters that they are certain about (Walter and Winterton 2007; Drinkwater 2009). One caveat of this approach is that it is time consuming to work through many characters (Stagg et al. 2015). However, algorithms giving continual preference to characters with the most discriminatory power, as is the case in Xper^3^, offsets this limitation to some degree (Walter and Winterton 2007; Drinkwater 2009). Furthermore, in challenging species groups, increased identification accuracy should arguably take preference over identification time. In the case of *Thesium* specifically, identification time using the interactive photographic key is unlikely to exceed identification time using the traditional textual keys provided by Hill (1915, 1925).

The electronic key further improves identification efficiency by subdividing particularly confusing and cryptic characters into more digestible units (Drinkwater 2009). During the reconstruction and revision of the identification key, this approach not only resulted in a more user-friendly key but also allowed for more precise character coding (Drinkwater 2009). For instance, the habit (growth form) of *Thesium* species, although often variable (Cohn 2004; Luo et al. 2012; Gamoun 2014), is an important distinguishing character. To improve the unambiguity of this valuable character, habit was divided into three separate characters namely, shape, woodiness and branching position. Furthermore, minimizing the number of character states that users are presented with at each character facilitated ease of use and decreased the chances of incorrect user interpretation.

**Character and character state presentation**

One of the main advantages of electronic identification keys when identifying species with cryptic characters is the illustration of characters using multiple aids, which greatly reduces ambiguity (Lawrence and Hawthorne 2006; Drinkwater 2009; De Vaugelas et al. 2011). Optimal visual presentation of each character and character state ideally requires sufficient photographs to illustrate the full range of variation, thereby leaving little to no room for user misinterpretation (see Kirchoff et al. 2011). For species with cryptic morphology, electronic key construction therefore goes hand in hand with field observations and photographs of live material. Unfortunately, in the majority of cases, acquiring the necessary photographs remains a major challenge due to resource and logistical constraints, especially in groups with many or rare species. Nevertheless, without adequate visual aids, the efficient and accurate identification of species with cryptic characters is improbable.

It is also true that images may contain only partial information (Joly et al. 2019) and should thus be supplemented with textual aids that are tailored to the requirements of the target audience. In the case of *Thesium* (and likely other species groups with cryptic morphology) the need for a user-friendly identification guide that can be used by both specialist and non-specialist users was immediately apparent. While generalist terminology saves non-specialist users the time and resources needed to familiarize themselves with the workings of a specific group, specialist terminology allows specialists to cross-reference information in the key with other taxonomic literature.

### Key evaluation

**Checkbase**

The Xper^3^ evaluation tool Checkbase showed that five species pairs were only partially discriminated: (1) *T. racemosum* Bernh. and *T. costatum* A.W.Hill, (2) *T. gracilarioides* A.W.Hill and *T. multiramulosum* Pilg., (3) *T. gracilarioides* A.W.Hill and *T. resedoides* A.W.Hill, (4) *T. gracile* A.W.Hill and *T. utile* A.W.Hill, and (5) *T. asterias* A.W.Hill and *T. ovatifolium* N.Lombard & M.M.le Roux. These species pairs are morphologically similar and the coding of multiple character states to account for variation resulted in partial, but not full, overlap in some characters. While this result highlights the challenge of successfully separating species with cryptic morphology using electronic keys (as well as traditional keys), these species can nonetheless be successfully identified using their respective species profiles as discussed before. All of the characters and character states included in the key provided full discrimination between species (as opposed to only partial discrimination or no discrimination). One exception was the Western Cape Province character state under the geographical distribution character, as none of the species included in the key occur in the province. It was, however, retained along with the other eight provinces of South Africa for completeness and to allow for future expansion in the scope of the key.

**Target group evaluation**

The target group evaluation indicated that the proposed key could be useful for identifying species with cryptic morphological characters and provided valuable suggestions for improvement that were subsequently incorporated. Differences in user interpretation of character states had to be addressed and subsequently, following Kirchoff et al. (2011) and Leggett and Kirchoff (2011), some arrows and/or circles were added. Furthermore, we replaced images causing uncertainty with superior images and incorporated labels.

During the evaluation, it was clear that some characters were problematic. Participants had very subjective interpretations of the degree of woodiness of plants (originally divided into herbs, suffrutices and shrubs) and consequently had trouble identifying the correct character state. To address this unambiguity, the number of character states was reduced to two: plants that were obviously herbaceous (including suffrutices) and robust woody plants. Corresponding textual descriptions were also revised and expanded, and clearer photographs were used to illustrate each character state. Similarly, the difficult-to-interpret inflorescence structure was simplified from six complex character states (e.g., monotelic racemose inflorescence with a terminal dichasial cyme, and simple or compound dichasial and monochasial cymes) to four, more general types (raceme-like, spike-like, cymes and solitary). The majority of participants were not able to utilize the placental column shape (generally < 2 mm) as they could not successfully dissect flowers to access this structure. Although there is little that can be done to improve this hurdle, the character was retained in the key as it is valuable for specialist use, and it is not crucial for species identification so that non-specialist users can simply forgo it.

The last method employed to improve the accuracy of the identification key was the coding of multiple character states (multiple correct answers) where necessary. This step is crucial as it accounts for intra-specific variation in characters, characters with continuous character states and also for user subjectivity (Drinkwater 2009; Stagg et al. 2015). For example, participants had difficulty determining the character state for corolla lobe hairs, partly due to user subjectivity and partly due to the fact that there is an almost continuous range of character states, from dense hairs to sparse hairs to papilla to smooth lobes. It is suggested that multiple character states are coded where a character state of a species is intermediate between two character states in the key, thereby resolving the problem of continuous characters, as well as subjective user interpretation. This flexibility in coding optimizes the chances of correct identifications without jeopardizing the discriminatory power of the key, as species are separated based on a combination of many characters. However, this approach should be applied conservatively to ensure that overall distinguishing power is not significantly reduced and that characters do not become redundant (see Jouveau et al. 2018). In Xper^3^, the key can easily be checked for redundancies using the item ‘comparison tool’, which indicates whether each character provides full discrimination, partial discrimination or no discrimination between species.

### Suggested best practices

Based on the pilot electronic identification key presented here, the following best practices are suggested for the unambiguous presentation of cryptic morphological characters and their character states in electronic identification keys: (1) maximization of the number of valuable characters; (2) minimization of the number of character states associated with each character; (3) division of difficult/complex characters into multiple simpler characters; (4) illustration of characters and character states using multiple aids such as visual and text descriptions; (5) illustration of character states using multiple photographs to show the entire range of variation (if applicable); (6) use of photographs of live material (as opposed to preserved material) and plants in situ where possible; (7) addition of labels and accents such as arrows or circles to photographs to highlight relevant characters; (8) tailoring text descriptions to the target audience(s) (generalist or specialist terminology, or both); and (9) coding for multiple character states (multiple correct answers) where intra-specific variation is present or if a species falls on or close to the border between two character states (to ensure that the discriminatory power of characters is not lost).

Other general best practices include: (10) ensuring sound taxonomy and clearly defined species concepts prior to key construction; (11) using software that allows for updates and improvements (as necessitated by user feedback and ongoing research), including the replacement of images with superior ones as they become available; (12) utilizing a multi-access key approach [as opposed to a single-access approach (dichotomous or polychotomous)]; (13) using species profiles with representative photographs and supplementary information including (14) photographs of diagnostic features and the general impression, size and shape (GISS); (15) detailed distribution maps (if species are geographically separated) and (16) diagnostic notes separating morphologically similar species; and (17) evaluation of proposed identification keys by participants from the target audience and the subsequent incorporation of feedback prior to publication.

## Conclusions

Electronic identification keys are valuable resources for species identification, which underpins all biological sciences. This study contributes to the rather limited body of knowledge on the successful identification of enigmatic species with cryptic morphologies using contemporary identification aids. It has shown that well-constructed electronic identification keys are feasible and offer the possibility of accurate identifications, in particular for species with cryptic characters, despite apparent contradictory reports in the literature. We have gained valuable insights into not only the problems and challenges associated with the successful identification of *Thesium* species (as a practical example of species groups with cryptic morphology) but also possible solutions and circumventions for difficulties in electronic key construction.

Ultimately a sound knowledge of the taxonomy and diagnostic characters of the taxa will determine the quality and efficacy of the identification key, regardless of the technology used in its construction and presentation. High attention to the presentation of the characters and their respective states are critical. There is no substitute for careful field studies of live organisms in their natural environment to overcome the typical limitations imposed by preserved specimens. This means a much greater effort in data collection but also a much greater reward in achieving a high level of discriminatory power in the identification key. Such electronic identification keys maximise the benefits that can be derived from the use of digital images and undoubtedly increase the accuracy of identification and reduce ambiguities that lead to a more user-friendly product for both specialist and generalist users. This might be especially valuable in economically important species groups such as grasses, which are characterised by cryptic morphological characters, by expanding the suit of potential users to farmers, conservationists, ecologists and so forth. The gap between research and users can also be minimised by adding the latest information on subjects such as synonyms, ecology and potential uses to species profiles.

To our knowledge, the best practices suggested here (although a combination of novel and previously known guidelines) are the first guidelines on electronic identification key construction tailored to species with cryptic morphology. While these guidelines work well for *Thesium*, similar studies of other species groups with cryptic morphologies will test these best practices, and likely reveal additional challenges and guidelines. This study therefore serves as a starting point for similar studies.
